# Externalizing as a common genetic influence for a broad spectrum of substance use and behavioral conditions: A developmental perspective from the Avon Longitudinal Study of Parents and Children

**DOI:** 10.1111/add.70163

**Published:** 2025-08-06

**Authors:** Wei Q. Deng, Kyla Belisario, Amanda Doggett, Marie Pigeyre, Guillaume Pare, Marcus R. Munafò, James MacKillop

**Affiliations:** ^1^ Peter Boris Centre for Addictions Research St. Joseph's Healthcare Hamilton Hamilton Canada; ^2^ Department of Psychiatry and Behavioural Neurosciences McMaster University Hamilton Canada; ^3^ Department of Medicine Faculty of Health Sciences, McMaster University Hamilton Canada; ^4^ Population Health Research Institute David Braley Cardiac, Vascular and Stroke Research Institute Hamilton Canada; ^5^ Thrombosis and Atherosclerosis Research Institute David Braley Cardiac, Vascular and Stroke Research Institute Hamilton Canada; ^6^ Department of Pathology and Molecular Medicine McMaster University Hamilton Canada; ^7^ School of Psychological Science University of Bristol Bristol UK; ^8^ MRC Integrative Epidemiology Unit University of Bristol Bristol UK

**Keywords:** ALSPAC, eating behavior, externalizing, gambling, pathways, polygenic risk scores, problematic internet use, self‐regulation, substance use

## Abstract

**Background and aims:**

Recent large studies have established the genetic basis of several conceptually linked phenotypes of externalizing. Polygenic risk scores (PRSs) for these constructs are associated with a range of substance use and mental disorder phenotypes but have not been examined with both pharmacological and non‐pharmacological addictive behaviors, or across a developmental window. This study identified biological pathways responsible for observed associations between PRSs and addiction phenotypes.

**Design, setting, participants:**

We selected genome‐wide association studies of 22 phenotypes, including substance use, general factors of externalizing and addiction, impulsivity and psychiatric conditions. Using summary statistics, we constructed PRSs in the offspring from the Avon Longitudinal Study of Parents and Children (ALSPAC) (n_max_ = 4995). Participants were genetically confirmed to be unrelated and of European‐like genetic similarity.

**Measurements:**

We examined the associations between PRSs and addiction‐related phenotypes including substance use, gambling, eating behaviors and internet use across different life stages, from adolescence to young adulthood. PRSs were partitioned by biological pathways to examine the common and unique mechanisms underlying the genetics of addiction‐related phenotypes.

**Findings:**

The PRS of externalizing factor (PRS_EXT_) showed the strongest association across phenotypes for substance use (minP = 2.6 × 10^‐31^, adjusted R^2^ = 0.10–4.72%), gambling (minP = 1.0 × 10^‐9^, adjusted R^2^ = 0.18–1.50%), eating behaviors (minP = 8.2 × 10^‐4^, adjusted R^2^ = 0.11–0.65%) and internet use (minP = 1.4 × 10^‐7^, adjusted R^2^ = 0.17–1.04%). Sensitivity analyses excluding a small subset of ALSPAC participants who also contributed to the externalizing summary statistics, yielded consistent association effect sizes (R^2^ = 0.98), suggesting minimal bias. The results also revealed several time‐varying associations between several PRSs and addiction phenotypes. Notably, the genetic influence of externalizing factor on alcohol and tobacco use was significantly stronger at younger ages. Finally, we identified multiple biological pathways that contribute to the link between addiction‐related phenotypes and PRS_EXT_, emphasizing the importance of synaptic functions and neuronal plasticity in the context of gambling and substance use.

**Conclusions:**

There appears to be genetic evidence implicating externalizing as a common mechanism of substance and behavioral addictive behaviors. These results support the shared genetic liability across substance misuse, problematic gambling and internet use, and demonstrate the potential utility of externalizing traits as a transdiagnostic dimension across diverse forms of psychopathology. Notably, the predictive power of externalizing genetic liability appears developmentally dynamic, supporting the view that externalizing represents a broad, non‐time‐invariant risk factor that may give way to more specific disorder‐related influences over time.

## INTRODUCTION

Self‐regulation is a psychological construct that broadly captures an individual's ability to control impulses and resist urges, which are considered mechanistic components for the development and maintenance of addiction [1, 2, 3, 4]. Difficulties in self‐regulation are often expressed as externalizing behaviors, such as risky substance use, aggression and hyperactivity, which are frequently associated with clinical conditions like attention‐deficit/hyperactivity disorder (ADHD) and substance use disorders (SUD). In the context of substance use, measures of self‐regulatory capacity (i.e. impulsive choice, impulsive action and impulsive personality traits) have been associated with both initiation and success of cessation [5, 6, 7, 8, 9, 10, 11, 12].

Remarkable progress has been made in understanding the genetic basis of addiction and related behaviors via genome‐wide association studies (GWASs). These findings fall into three domains: (1) drug or syndrome‐specific GWASs (e.g. for substance use or problematic use); (2) broader behavioral outcomes (e.g. externalizing, internalizing, addiction factor); and (3) specific psychological processes related to impulsivity (e.g. delay discounting, Urgency‐Premeditation‐Perseverance‐Sensation seeking‐Positive urgency; UPPS‐P). Specifically, meta‐analyses of GWASs on alcohol [13, 14, 15, 16], nicotine [17, 18], cannabis [19, 20, 21, 22, 23] and opioid use [24, 25] have yielded a large number of novel loci, for which many have been shown to generalize across populations. However, large‐scale studies have limitations, such as the traits are often measured using broad, standardized tools that may not fully capture the nuances of the condition, leading to potential misclassification or dilution of the trait being studied. Further, although associations were identified, the insights provided were often on substance‐specific biology.

Leveraging the shared genetic liability, GWASs of seven related phenotypes (i.e. ADHD, problematic alcohol use, lifetime cannabis use, age at first sexual encounter, number of sexual partners, risk tolerance and age of smoking initiation) were combined in a genomic structural equation model (gSEM) [26] to identify genetic loci that contribute to a common factor for externalizing [4]. The polygenic risk score (PRS) for the genomic factor of externalizing factor (PRS_EXT_) has been robustly linked to substances misuse and risky behaviors beyond the seven phenotypes in the original analysis, indicating a broad influence on behavioral patterns. The gSEM approach also yielded a general addiction risk factor (addiction‐rf), combining problematic alcohol and tobacco use, cannabis use disorder and opioid use disorder [27], which has been shown to capture genetic liability to substance addiction, irrespective of the substance.

Recent GWASs have also demonstrated the genetic influence on specific psychological processes of impulsivity [28, 29, 30, 31, 32], as captured by delay discounting [33], the UPPS‐P Impulsive Behavior Scale [34] and the Barratt Impulsiveness Scale [35]. Although the GWAS discovery sample size for these traits is comparatively smaller than other domains (e.g. the largest sample size is 133 517 vs. millions for substance use), there is notable overlap in gene signals (e.g. *CADM2, TCF4* and *NCAM1* genes) and overall genetic correlation. For example, genetic correlation between externalizing factor and delay discounting was reported at 0.311. Regardless of the sample size differences, genetic variants identified from these GWASs have been consistently linked to a common set of traits, including risky substance use, psychiatric disorders characterized by a high level of impulsivity, a higher body mass index (BMI) and a low level of subjective well‐being [4, 32, 36, 37, 38].

This premise holds the potential for a broader application, suggesting that previously identified genetic influences may be examined in a wider spectrum of non‐substance‐based addictions (i.e. behavioral addictions). Behavioral addictions, like gambling or problematic internet use, also exhibit a pattern of compulsion and persistence despite negative consequences [39, 40, 41], similar to those seen in SUDs. Emerging research indicates that genetic factors may also contribute to non‐substance‐related addictions, such as disordered gambling [42, 43], binge eating [44, 45] and problematic internet use [46, 47], albeit significant associations for disordered gambling have not yet been identified [42, 43]. This set of GWASs provided evidence that non‐substance use addictions may share a common genetic basis with psychiatric disorders, including ADHD, bipolar disorder, major depression disorder and schizophrenia (SCZ) [45, 47], but direct genetic links with mechanisms of addiction beyond psychiatric conditions have not been explored.

An alternative is to evaluate genomic correlates through PRSs constructed from well‐powered GWASs. This approach has demonstrated the genetic links between disordered gambling and SCZ [48], between the big five personality traits (openness, conscientiousness, extraversion, agreeableness and neuroticism) and binge eating [49], as well as between bipolar disorder and BMI [50, 51]. Although these findings offer valuable clues about the potential common genetic influences on comorbid behavioral addictions, they principally reinforced existing epidemiological links without leveraging information within the PRSs to understand the underlying biological mechanisms. Another consideration is the progression of addictive behaviors over time, which may be variably driven by genetics or exhibit temporal trends [52, 53]. Therefore, we propose to address these gaps by conducting a phenome‐wide evaluation of substance and non‐pharmacological addictive behaviors using PRSs at various developmental stages and partitioning PRSs by meaningful molecular pathways.

The current investigation explores the common genetic basis across addiction phenotypes (non‐substance and substance‐related) in offspring from the Avon Longitudinal Study of Parents and Children (ALSPAC), a longitudinal cohort study with genomic data and diverse health outcomes across the lifespan. First, we examined associations between PRSs and phenotypes in four broad categories of substance use, gambling, internet use and eating behaviors over different life stages, from early adolescence to young adulthood. Second, we focused on PRSs linked to multiple categories to pinpoint shared or unique genetic influences and identified biological pathways responsible for the observed associations between these PRSs and addiction phenotypes.

## METHODS

### Target study sample overview

The current study is based on the genetic subset of children from ALSPAC, a United Kingdom (UK)‐based birth cohort [54, 55, 56]. The study originally invited pregnant women living in Avon, United Kingdom with expected dates of delivery between 1 April 1991 and 31 December 1992, to take part in the study; 20 248 pregnancies were identified as being eligible and the initial number of pregnancies enrolled was 14 541. These mothers (Generation 0) and children (Generation 1) provided consent for the collection of genetic and phenotype data. Please note that the study website (http://www.bristol.ac.uk/alspac/researchers/our-data/) contains details of all the data that is available through a fully searchable data dictionary and variable search tool. Data from age 22 onward were collected using REDCap [57].

### Phenotypic categories

We curated variables on substance use, gambling, eating behaviors and internet use. There were four distinct combustible tobacco phenotypes: age of initiation, current use status and quantity of use and dependency measured by the Fagerström Test For Nicotine Dependence (FTND) [58]; four e‐cigarette phenotypes: age of initiation, current use status, frequency, quantity (milliliters of vaping fluid); two alcohol phenotypes on quantity‐frequency and alcohol‐related problems based on the frequency and severity sub‐scale scores of the Alcohol Use Disorders Identification Test (AUDIT) [59]; and four cannabis phenotypes for the age of initiation, current use status and frequency and dependency measured by the Cannabis Abuse Screening Test (CAST) [60]. Age of initiation was consolidated across waves to minimize missingness because of earlier non‐endorsement of initiation. Gambling frequency questions (e.g. horse race, scratch cards, lottery, *etc*.) and the 9‐item Problem Gambling Severity Index, extracted from the 31‐item Canadian Problem Gambling Inventory [61], were collected at 18, 20 and 24 using self‐completion questionnaires for their behaviors in the previous year. Disordered eating behaviors in the previous year were assessed at four time points at the ages of 13, 15, 18 and 24, for which the age 13 assessment was completed by mothers. The questions were based on a modified version of the Youth Risk Behavior Surveillance System questionnaire [62], independently validated in a sample of adolescents from the Growing Up Today Study [63]. To assess differences in PRS associations, we purposefully included what were considered ‘externalizing’ (i.e. binge eating, purging, excessive exercise and fasting) and ‘internalizing’ (i.e. self‐perception of body image) eating behaviors, and professionally diagnosed binge eating disorder, anorexia nervosa, bulimia nervosa at age 24. We retained the limited questions available on the number of hours spent on‐line per week concerning activities from information search, socializing, leisure and e‐commerce at ages 18 and 24, as well as the frequency of gaming and parental restriction because of internet use at age 13. The complete lists of variables are given in Tables S1 and S2, respectively. This yielded a total of 115, 139, 9 and 85 unique variables across the age range of 13 to 28 for eating behaviors, gambling, internet use and substance use, respectively (Table S3). Details on data selection and curation are available in Supporting information.

### Discovery GWAS samples

We obtained summary statistics from 22 large GWASs (Table S4): the genetic factors of externalizing [4] and general addiction [27]; three personality traits: adventurousness, risk tolerance [38], neuroticism [64]; six impulsivity measures: delay discounting [31], the 5‐factor impulsive personality measured by UPPS‐P [30]; six substance use phenotypes: smoking cessation, initiation, cigarettes per day [65], alcohol use disorder (AUD) [16], cannabis use disorder (CUD) [23] and opioid use disorder (OUD) [24]; and five psychiatric conditions: ADHD [66], bipolar disorder [67], major depressive disorder (MDD) [68], posttraumatic stress disorder (PTSD) [69] and SCZ [70] that were known to overlap in genetic architecture with substance use.

### Genetic data quality control, PRS construction and association analysis

Genetic data quality control for the 8932 Generation 1 samples with genome‐wide genotyping data has been described previously [71]. Briefly, imputation was done using the TOPMed‐r2 reference panel [72]. Imputed data were processed to retain unique bi‐allelic single nucleotide polymorphisms (SNPs) satisfying: r^2^ threshold > 0.3, minor allele frequency (MAF) > 0.01, genotype missing rate < 0.05 and Hardy–Weinberg disequilibrium *P* > 5 × 10^‐7^. We removed individuals with mismatched self‐reported and inferred genetic similarity group. The final data included 7975 unrelated European participants with 9 248 678 autosomal SNPs. PRSs were derived using Lassosum [73] for its robustness to misspecification of linkage equilibrium and the option to perform pseudo‐validation. Each PRS was pseudo‐validated or validated using the exact phenotype in ALSPAC as in the discovery GWAS, with weights applied to the full genetic sample and standardized to have a mean of zero and unit variance. For more details, please refer to the Supporting information. PRSs that were significantly associated with target or proxy phenotypes after multiple hypothesis corrections via the Benjamini‐Hochberg procedure [74] were retained. A linear regression model was used to test the association between each pair of retained PRS and addiction phenotype, adjusting for genetic sex, age at phenotype collection (except for age of initiation) and the first 10 genetic principal components (PCs). For each phenotype, the amount of variance explained by PRS was calculated as the difference in adjusted R^2^ between the full model (PRS and all covariates) and the model with only covariates. A *q*‐value < 0.05 accounting for all phenotypes tested within each PRS was deemed statistically relevant for further interrogation. We also examined PRS × sex interaction by testing whether the interaction term was significant in addition to the PRS and the sex main effects. To evaluate the PRS × sex interaction effects, we also examined another robust model including all covariate × PRS and covariate × sex and/or age interactions in the regression model as additional covariates to reduce bias in effect estimates [75]. We considered a PRS × sex interaction significant only if both the main PRS effect and the interaction term from the robust model passed an false discovery rate (FDR)‐corrected threshold of <0.05. Additionally, because ALSPAC contributed to the externalizing GWAS (n_max_ = 1660), we performed a sensitivity check by removing these participants (24%–44% reduction) from the PRS_EXT_ association analysis to assess potential bias [76, 77]. All statistical analyses were conducted in Statistical Software R version 4.1.0 [78]. As a sensitivity analysis, we compared the sample size and association strength of phenotypes in each category as a function of statistical significance (*q*‐value < 0.05 vs. otherwise). The study analysis was not pre‐registered, and therefore, results should be considered exploratory.

### Trends in PRSs association across development

A subset of addiction phenotypes was collected at multiple time points. Therefore, we modeled the strength of their PRS‐phenotype associations in terms of adjusted R^2^ as a function of the average age of the participants. The best‐fitted line was estimated using the generalized additive models (GAM) with smoothness determined by 3 (or 2) degrees of freedom for phenotypes with more than 3 (or 2) time points. The statistical significance of the smooth terms was evaluated using *F*‐ratio comparisons with a parametric component for linear relationships and a non‐parametric component for non‐linear relationships [79].

### Partitioning PRSs by enriched pathways

For PRSs with some explanatory power (>1% variance explained) and showing consistent associations (*q*‐value < 0.05) with addiction phenotypes across all categories, we partitioned the genetic effects within the established PRS to identify biological pathways that contributed to the observed associations. Specifically, we focused on SNPs with non‐zero weights in the PRS and then mapped these SNPs to the nearest genes (+/− 100 Kb). The mapped genes were then used to test for enrichment in Kyoto Encyclopedia of Genes and Genomes pathways [80] using an FDR‐adjusted *P*‐value cut‐off of 0.01, producing pathways in seven broad categories, namely, metabolism, genetic information processing, environmental information processing, cellular processes, organismal systems, human diseases and drug development. We, then, partitioned the PRS by enriched pathways: a pathway‐specific score (i.e. partitioned PRS) was calculated using only SNPs assigned to that pathway. Note, that since the same SNP can be mapped to multiple genes/pathways, each SNP (through genes) can contribute to more than one pathway‐specific PRS. These pathway‐specific PRSs were then tested for association with all phenotypes as described previously, regardless of whether the association with the overall PRS was significant or not.

## RESULTS

### Sample characteristics and curated phenotypes

A summary of key phenotypes across categories at different life stages can be found in Table S5. The missing data in our study primarily resulted from the design of data collection across different subsamples, rather than from non‐response or failure to report. While these data points are technically ‘missing,’ (and are listed as such in Table S5 for transparency), they are missing by design, rather than as a result of attrition or non‐response. For each variable, data were collected approximately at the same time frame, therefore, differences in the participants' ages were minimal. Using the problematic or moderate risk criteria for substance use, we found the overall severity to be relatively low in this cohort (Table S5).

There were considerable within‐category correlations for substance use, internet use, gambling and eating behavior phenotypes (Figure S1). In particular, there was a broad pattern of negative correlation between e‐cigarette use and other substances (except cigarette smoking). Similarly, while cannabis use was positively correlated with alcohol use phenotypes, it also demonstrated negative correlations with both cigarette and e‐cigarette smoking phenotypes. Gambling phenotypes were generally positively correlated with each other, with the minimal pairwise correlation at −0.08. In contrast, individual eating behaviors had a more balanced pairwise correlation ranging from −0.62 to 0.82. Parental restrictions were negatively correlated with internet use frequency, but frequencies of various internet use activities were generally positively correlated, except ‘information searching (e.g. for school or work, looking up news)’.

### Properties of the derived PRSs

There were 20 PRSs significantly associated with their target or proxy traits in ALSPAC (Table 1). Note that as there were only two cases of SCZ and a low count of participants who ever used opioids, we examined PRS_OUD_ and PRS_SCZ_ with the CAST score and a depression diagnosis, respectively. Generally, PRSs derived from well‐powered GWASs had good predictive performance. Of particular note, PRS_EXT_, PRS_Adventurousness_, PRS_MDD_ and two PRSs of impulsivity, lack of premeditation (PRS_UPPS‐PreMed_) and sensation seeking (PRS_UPPS‐SS_) explained between 1.05% and 3.25% of the phenotypic variance (Table 1). The amount of explained variance was noteworthy given the reported SNP‐heritability for these traits ranged from 5 to 10%. We also observed similar correlation patterns between PRSs and reported genetic correlations (Figure S2). In addition, PRS_EXT_, PRS_addiction‐rf_ and PRS_MDD_ showed a broad pattern of correlations with the other PRSs (Figure S2).

**TABLE 1:** Performance and properties of the 22 PRSs in ALSPAC.

PRS construction and validation using Lassosum	Lassosum PRS properties			In‐sample performance					Included in PheWAS (FDR *q* < 0.05)
	No. of SNPs matched	No. of SNPs with non‐zero weights	Lassosum option	Validating phenotype or a proxy	Validation sample size	*P*‐value	*Q*‐value	Adjusted R^2^ (%)	
Delay discounting	7 614 379	4 465 424	Validation with phenotype	Monetary Choice Questionnaire derived DD at YPH (28 y)	2855	6.58E−03	8.04E−03	0.22	Yes
Lack of premeditation (UPPS‐P)	7 407 059	4 575 231	Validation with phenotype	Lack of premeditation at YPH (28 y)	2941	4.39E−07	9.66E−07	0.83	Yes
Lack of perseverance (UPPS‐P)	7 407 059	761 445	Validation with phenotype	Lack of perseverance at YPD	2946	1.35E−08	5.96E−08	1.05	Yes
Positive urgency (UPPS‐P)	7 407 059	4 579 516	Validation with phenotype	Positive urgency at YPD	2945	6.21E−08	1.95E−07	0.95	Yes
Negative urgency (UPPS‐P)	7 407 059	4 602 030	Validation with phenotype	Negative urgency at YPD	2949	2.68E−07	6.54E−07	0.86	Yes
Sensation seeking (UPPS‐P)	7 407 059	4 610 635	Validation with phenotype	Sensation seeking at YPD	2942	6.19E−13	4.54E−12	1.53	Yes
Addiction‐rf	3 493 489	14 381	Pseudo‐validation	AUDIT‐P at YPH (28 y)	2670	2.38E−05	3.73E−05	0.63	Yes
Externalizing	6 133 431	34 154	Pseudo‐validation	Age when YP first smoked a cigarette—consolidated across waves.	3450	3.90E−27	8.57E−26	3.25	Yes
Adventurousness	870 462	378 975	Validation with phenotype	Sensation seeking at YPH	2942	1.42E−22	1.56E−21	2.82	Yes
General risk tolerance	7 295 105	428 183	Validation with phenotype	UPPS‐P item 9: YP quite enjoys taking risks YPH (28 y)	2986	2.34E−08	8.57E−08	0.97	Yes
Neuroticism	7 016 355	33 158	Pseudo‐validation	Neurotic symptom score at F17 (17 y)	1757	2.25E−05	3.73E−05	0.96	Yes
Cigarettes per day	2 100 127	73 789	Validation with phenotype	No. of cigarettes YP smokes per day, on average YPD (24 y), with imputed zeros for those do not smoke.	2980	5.62E−06	1.03E−05	0.66	Yes
Smoking cessation	2 092 178	53 793	Pseudo‐validation	No. of cigarettes YP smokes per day, on average YPD (24 y), with imputed zeros for those do not smoke	2980	1.04E−03	1.43E−03	0.33	Yes
Smoking initiation	2 099 285	20 411	Pseudo‐validation	Age when YP first smoked a cigarette—consolidated across waves.	3450	9.04E−08	2.49E−07	0.79	Yes
Alcohol use disorder	5 379 095	130 253	Validation with phenotype	AUDIT‐P at YPH (28 y)	2670	6.54E−07	1.31E−06	0.88	Yes
Cannabis use disorder	8 746 529	3 120 546	Pseudo‐validation	The Cannabis Abuse Screening Test score at 24 y	2733	0.41	0.43	0	No
Opioid use disorder	3 262 923	6428	Pseudo‐validation	Age when YP first smoked a cigarette—consolidated across waves.	3450	0.0072	0.0084	0.18	Yes
ADHD	6 641 394	21 470	Pseudo‐validation	Lack of premeditation at YPH (28 y)	2941	1.89E−03	2.45E−03	0.29	Yes
Bipolar disorder	71 999 011	31 293	Pseudo‐validation	Neurotic symptom score at F17 (17 y)	2761	0.71	0.71	0	No
Major depressive disorder	6 356 238	19 869	Pseudo‐validation	Ever been diagnosed with depression—YPB (22 y)	2761	1.14E−10	6.27E−10	1.45	Yes
Posttraumatic stress disorder	9 289 653	36 085	Pseudo‐validation	Respondent has ever experienced any other very traumatic or extremely stressful event	2752	0.033	0.036	0.13	Yes
Schizophrenia	7 282 571	32 862	Pseudo‐validation	Ever been diagnosed with depression—YPB (22 y)	2761	4.07E−04	5.98E−04	0.41	Yes

Abbreviations: ADHD, attention‐deficit/hyperactivity disorder; ALSPAC, Avon Longitudinal Study of Parents and Children; AUDIT, Alcohol Use Disorders Identification Test; DD, delay discounting; FDR, false discovery rate; PheWAS, phenome‐wide association study; PRS, polygenic risk score; SNP, single nucleotide polymorphism.

### Phenome‐wide association with PRSs

The phenome‐wide association results revealed a larger set of overlapping PRSs that were associated with substance use and gambling phenotypes (Figure S3–S4; Table S6), including PRS_EXT_, PRS_addiction‐rf_, PRS_SI_ (PRS for smoking initiation), PRSs for substance use disorders (opioid, cannabis, alcohol use) and psychiatric conditions (ADHD, MDD and PTSD). While the available sample size was similar across phenotypes in the eating behavior and internet use categories, significant results in the other two domains were driven by phenotypes with more samples (Figure S5). Of the 363 phenotypes across four categories spanning as many as 14 unique time points, 195 phenotypes (54%) were associated with at least one of the 20 PRSs. In particular, PRS_EXT_ and PRS_ADHD_ were associated with the highest number of phenotypes, 109 and 124, respectively.

**FIGURE 1:**
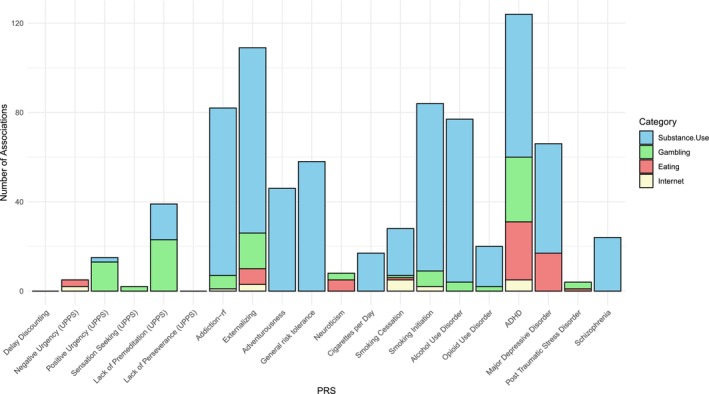
Summary of associations across phenotypic categories. Bar plots displaying the number of associations across four categories (substance use, gambling, eating behaviors and internet use) for 20 polygenic risk scores (PRSs) significantly associated with their corresponding traits or proxies. The height of each bar represents the total number of associations for a given PRS, with different colors indicating the contribution of each category to the overall total. The y‐axis denotes the number of associations, and the x‐axis lists the individual PRSs.

**FIGURE 2:**
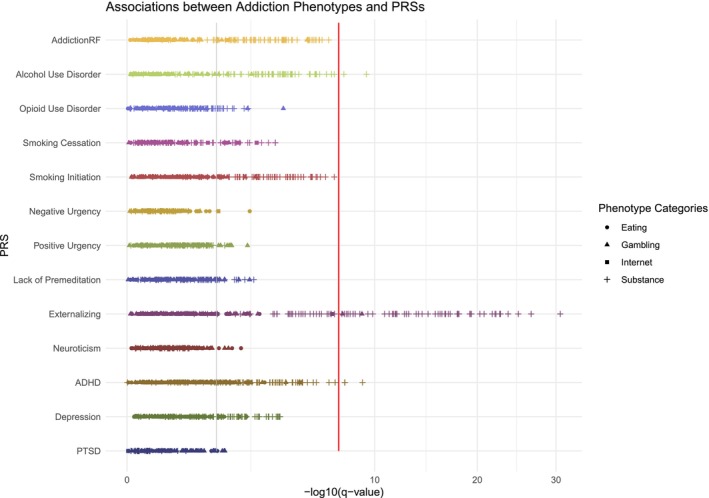
A summary of phenome‐wide associations with significant polygenic risk scores (PRSs). We visualize the association strength (‐log10 of *q*‐value) between 13 PRSs associated with phenotypes from at least two categories and all addiction‐related phenotypes (as dots of different shapes). The x‐axis is the ‐log10 of the false discovery rate (FDR)‐adjusted *P*‐value to indicate the strength of each association, with higher values denoting more significant associations. The y‐axis represents different PRSs. Phenotypes for eating behavior, gambling, internet use and substance use are indicated by a circle, triangle, square and a plus. The vertical lines at *y* = ‐log10(0.05) and *y* = ‐log10(5 × 10^‐8^) are included to indicate thresholds for different levels of statistical significance, with higher values representing stronger evidence.

Most notably, PRS_EXT_, PRS_ADHD_ and PRS for smoking cessation (PRS_SC_), were associated with phenotypes across all four categories (Figure 1). Additionally, 10 PRSs were associated with phenotypes from at least two categories, including PRS_addiction‐rf_, PRS_SI_, PRS_SC_, PRS_neuroticism_, PRS_AUD_, PRS_OUD_, PRS_MDD_, PRS_UPPS‐PreMed_ and PRSs for positive urgency (PU) and negative urgency (NU), or PRS_UPPS‐NU_ and PRS_UPPS‐PU_, respectively. We found that PRSs of substance use were often associated with phenotypes from substance use and gambling categories, whereas PRS_MDD_ and PRS_neuroticism_ were linked to substance use and eating behavior phenotypes. PRSs related to impulsivity showed specificity to variables in the gambling category (Figure 1). Figure 2 summarizes the strength and categories of phenotypic associations for these 13 PRSs, where PRS_EXT_ had the strongest association signals overall. Besides PRS_EXT_, the strongest predictors for gambling frequency were PRS_OUD_, PRS_ADHD_ and PRS_addiction‐rf_ (Figure 2). Meanwhile, PRS_MDD_, PRS_ADHD_ and PRS_neuroticism_, were strongly associated with eating behaviors (Figure 2). Unlike gambling and substance use phenotypes, eating behaviors (57/115) were only linked to a handful of PRSs. While the majority of phenotypes were uniquely associated with PRS_ADHD_ and PRS_MDD_, we also observed partial overlaps in associated eating behaviors between PRS_ADHD_/PRS_EXT_ and PRS_MDD_ and between PRS_MDD_ and PRS_neuroticism_ (Figure S5). Interestingly, PRS_ADHD_/PRS_EXT_ was associated with actions directly related to eating, such as exercising, skipping meals or making oneself sick to avoid putting on weight (Table S7), whereas PRS_MDD_/PRS_neuroticism_ was uniquely associated with negative feelings associated with consequences of binge eating, such as ‘afraid of gaining weight or getting fat’ or ‘upset or distressed about weight/body shape’.

The sensitivity analysis in a subset of n_max_ = 3440 participants produced materially the same results for PRS_EXT_ —all effect sizes overlapped within the 95% CI of the original effect sizes, and the linear regression comparing estimates before and after exclusion explained almost all variation (Figure S6) (R^2^ = 0.98). This suggests any bias from sample overlap was minimal.

We also found PRS × sex interactions to be specific to substance use and gambling, with significant main and interaction effects (both *q* < 0.05) from PRS_EXT_, PRS_DD_, PRS_UPPS‐PU_, PRS_UPPS‐PreMed_, PRS_UPPS‐SS_ and PRS_Adventurousness_, explaining between 0.28% and 1.53% of the phenotypic variance (Table S8).

### Phenome‐wide association with PRSs across development

**FIGURE 3:**
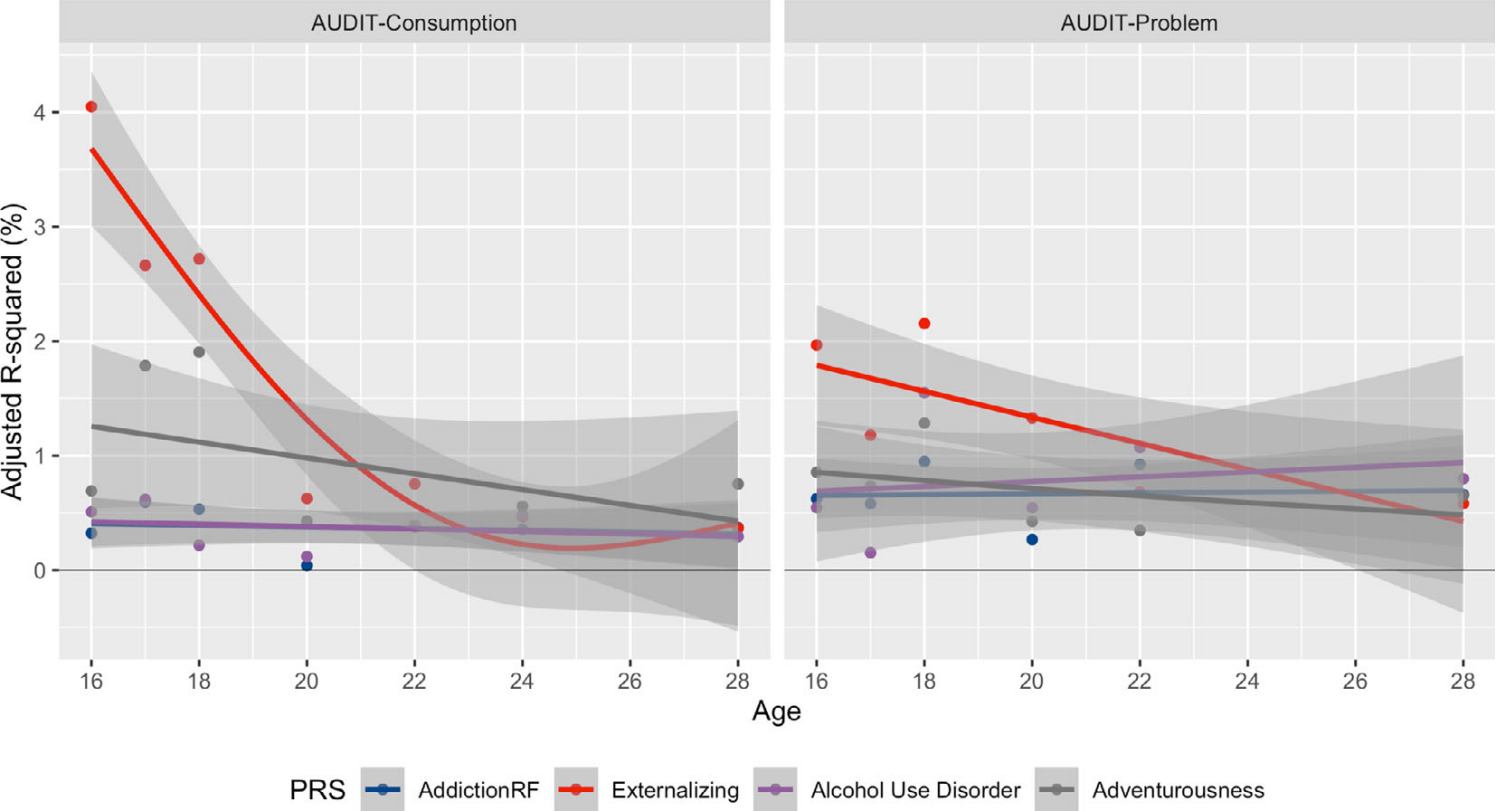
A longitudinal trend of associations between selected polygenic risk scores (PRSs) and alcohol phenotypes at multiple time points. The association strength (adjusted R^2^) is shown as a function of the participant age when the phenotype was measured (ages 16 and 28). The left and right panels corresponded to results based on Alcohol Use Disorders Identification Test (AUDIT)‐C and AUDIT‐P. Each dot represented the estimated adjusted R^2^ using the PRS_addiction‐rf_, PRS_EXT_, PRS_AUD_ and PRS_Adventurousness_. The best‐fitted line was estimated using the generalized additive models with smoothness determined by 3 degrees of freedom. The shaded area around the fitted line represents the 95% CI, estimated using bootstrap sampling techniques.

Although PRS_EXT_ was associated with addiction phenotypes from all categories, the strongest signals were from cannabis use status at age 15 (*P* = 5.9E−33; adjusted R^2^ = 3.55%) (Table S6), AUDIT‐C at age 16 (*P* = 5.6E−29; adjusted R^2^ = 4.25%) and age of initiation for cigarette smoking (*P* = 3.9E−27; adjusted R^2^ = 3.25%). The genetic influence of externalizing factor on substance‐related phenotypes appeared stronger at younger ages, with a longitudinal trend observed for the association with alcohol consumption and severity (Figure 3). The strength of associations between the PRS_EXT_ and AUDIT‐C diminished over time from age 16 to 28 years (Table S9), and similarly for PRS_adventurousness_. In contrast, results for AUDIT‐P were consistent with a constant genetic influence over time (trend *P* > 0.1) (Table S9). In addition, there was a linear trend for cannabis use and the severity score (linear trend *P* < 0.05) (Table S9), whereby more variation in both use status and severity was explained by PRS_risk_ as participants aged (Figure S7). Finally, a non‐linear relationship was observed for smoking status concerning PRS_SI_ and PRS_ADHD_ (non‐linear trend *P* < 0.05) (Table S9), indicating changes in the genetic influence that varied with time (Figure S8). Longitudinal patterns were not significant for the remaining phenotypes, perhaps because of the relatively fewer time points and weaker associations (Figures S9–S10).

### Partitioning PRS_EXT_ and addictive behavior associations by enriched pathways

Although three PRSs were significantly associated with phenotypes from all four categories, only PRS_EXT_ consistently showed strong explanatory power (adjusted R^2^ > 1%) (Table S6). PRS_EXT_ included 34 153 SNPs (Table 1), mapping to 9446 unique Ensembl gene IDs. We identified 54 pathways for PRS_EXT_ (Table S10) in five broad categories, particularly in signal transduction (12/54), endocrine (11/54) and nervous systems (7/54). The most significantly enriched pathway was axon guidance (enrichment *P* = 8.1E−11).

Of these, 21 were associated with phenotypes in at least one category (Figure S11). The amount of phenotypic variance explained by the partitioned pathways ranged from 0.01% to 0.59% in contrast to 0.10% to 4.72% by the full PRS_EXT_. A large number of these pathways (16/21) were significantly associated with cannabis use (Figure S12). In particular, the retrograde endocannabinoid signaling pathway was linked to multiple cannabis and AUDIT traits between 15 and 18 (Table S11). Pathways relevant to non‐substance‐use traits included the Hippo signaling pathway, which regulates organ size and cell proliferation, and was associated with behaviors like tobacco smoking severity and ‘doing other things to avoid weight gain’. The parathyroid hormone pathway, crucial for calcium regulation, was linked to ‘strong desires for food’ and ‘concern about weight’. The Rap1 pathway, which is involved in cell adhesion, cell junction formation and cell polarity was the only pathway implicated in gambling behavior, and it overlapped with tobacco, cannabis use and internet use (Figure S12).

## DISCUSSION

In this study, we hypothesized that diverse PRSs, broadly reflecting self‐regulatory capacity, would be associated with a broad spectrum of addiction‐related phenotypes of substance use, gambling, eating behaviors and internet use in the ALSPAC cohort. Our findings strongly support the genetic contribution of externalizing factor to both substance‐ and behavioral‐based addiction. Notably, some of these genetic influences were time‐varying, suggesting externalizing genetic liability is developmentally dynamic—more impactful during adolescence, but becoming less predictive in adulthood as environmental and disorder‐specific genetic factors gain prominence. This also implies that providing additional support for youth during this disproportionally risky developmental window may reduce the expression of biological predisposition to adverse outcomes and vice versa. While extant GWASs show broad externalizing genetic associations mostly limited to substance disorders, our study is among the first to model multiple substance use, use disorders and behavioral addiction phenotypes within the same developmental cohort. Results from this study support the genetics of externalizing as a nexus that links both behavioral and substance‐based addictions.

Our results were supported by the careful choice of powerful genetic instruments, from a wide range of substance use, behavioral phenotypes and psychiatric conditions GWASs to elucidate the genetic overlap between substance and behavioral addictions. Behavioral addictions such as food addiction, problematic gambling and internet use are increasingly recognized as clinically relevant conditions and share key neurobiological and psychological mechanisms with substance‐based addictions. Pathological gambling was previously classified as an ‘Impulse‐Control Disorders Not Elsewhere Classified’ under the fourth edition of the Diagnostic and Statistical Manual of Mental Disorders (DSM)‐IV. Given its comorbidity with and other similarities to SUD, gambling disorder has since been added to the substance disorder chapter in the DSM‐5 [52, 81, 82]. Our results align with this re‐classification, revealing shared genetics with externalizing, impulsivity, multiple SUDs and psychiatric conditions. These results also highlighted the possibility of using genetically predicted externalizing factor to explain common co‐morbidities of problematic gambling and to potentially define subtypes of pathological gambling along with other well‐established risk factors, including age, education, marital status and financial well‐being [83]. Meanwhile, we found both internalizing (i.e. depression, neuroticism) and externalizing (i.e. ADHD and externalizing factor) genetic influence on eating behaviors, but pointing to slightly different subsets of eating‐related phenotypes. By situating these behaviors within a broader conceptual framework of addiction and impulsivity, we contribute to an integrated understanding of how different forms of dysregulated behavior may reflect common underlying vulnerabilities.

We honed in on addiction‐related phenotypes collected at multiple time points (ages 13–28) to understand the genetic influence over time. We observed time‐varying trends from the PRS_EXT_ (and several others) across categories of substances and behavioral phenotypes. For example, on average, there was an increasing genetic contribution to cannabis use and severity when participants were older (28 years vs. 15 years), but a decreasing contribution to frequency and other substance use phenotypes. These opposing trends could be because of the use of certain substances becoming less about deficits of self‐control and more about environmental and lifestyle factors, over time. For example, alcohol and smoking are more socially acceptable and available to young adults (18+) versus adolescents [16, 17, 18]. Conversely, because cannabis remains illegal in the United Kingdom, its use is inherently riskier, leading to a stronger association with risk tolerance and co‐use with alcohol. For gambling and e‐cigarette phenotypes, genetic contribution of PRS_EXT_ was mostly constant, but these were only measured at later time points (18+). Collectively, these suggest externalizing psychopathology to be a central construct that underlies high‐risk behaviors, particularly at early ages [84] and highlight the dynamic interplay between genetic predispositions to impulsivity and the environmental contexts that either exacerbate or mitigate these tendencies. This also aligns with the developmental models of psychopathology from twin studies [85, 86, 87, 88, 89], suggesting that broad, non‐specific liability gives way to more specific expressions of risk over time.

PRS × sex findings indicated a stronger genetic predisposition for substance use and gambling in males. It has been suggested that certain environmental factors tend to exert more influence on women compared to men [90], including social norms and expectations, pressures related to appearance and caretaking, all of which can lead to heightened stress, anxiety and behaviors like substance use or gambling as coping mechanisms [91]. No PRS × sex interaction was observed for eating behaviors, which seemed contrary to the widely reported sex differences in eating disorders. These could suggest that the genetics of self‐regulation might not impact eating behaviors differently between sexes or that the observed sex differences could either be because of genetic effects specific to eating pathology [92, 93], for example, via sex hormones [94] or environmental effects [94], such as increased societal/peer pressure for thinness among girls and women. Future studies using refined phenotyping and sex‐stratified GWAS that includes sex chromosomes may better uncover sex‐dependent risk pathways.

Decomposing PRS_EXT_ into biological pathways enabled us to examine the functional links that contributed to the observed associations between PRSs and multiple categories of addiction phenotypes. This approach represents an alternative avenue to generate biological insights for behavioral phenotypes currently less studied in GWASs. This collection of implicated pathways includes a mix of neural signaling, cell adhesion and reward‐processing systems. In particular, processes such as dopaminergic synapse, endocannabinoid signaling and GABAergic synapse showed specificity to substance use [95, 96, 97, 98, 99, 100, 101, 102, 103, 104, 105, 106, 107], while others such as focal adhesion, axon guidance and Rap1 signaling pathway, may relate more to structural changes and neural development, which are also key in nicotine and cocaine addiction [108, 109].

Our findings should be interpreted with limitations in mind. First, substance use phenotypes exhibited stronger and more consistent PRS associations. This could partly be because of better phenotyping as there is abundant literature on what measurements are considered trait‐like. Another contributing factor is statistical power, the genetics of substance use are more established than eating disorders and gambling, with better characterized biochemical processes and pathways from biobank‐scale GWASs. Second, ALSPAC contributed data to two of the seven GWASs (for cannabis use and smoking) that were incorporated into the GWAS of externalizing via the gSEM model. Several studies have demonstrated that sample overlap between discovery and target datasets can bias PRS association estimates [76, 77]. However, sample overlap between the externalizing GWAS and the present analysis was limited to 1660 participants (~0.12% of the total externalizing GWAS sample), and we have shown the resulting bias was minimal. Third, this study focused on individuals of the European genetic similarity group, and replication across diverse genetic backgrounds is needed as additional GWAS and samples become available. Further, we would like to point out what is considered problematic or ‘appropriate’ addictive behaviors can change in other samples or populations. For example, genetically predicted externalizing factor would unlikely predict smoking in South Asian women with a low prevalence estimated at 3.25% [110]. Similarly, should policy or social acceptability of cannabis relax the strength between genetically predicted externalizing factor and cannabis use would likely reduce at younger ages. Finally, the reported effect size should also be interpreted with the available sample size for each measure at each time point as not all measures were assessed at all time points in ALSPAC, therefore, there is intermittent missingness that modestly impacts the sample size depending on the measure and time it was assessed.

To conclude, we established the genetic evidence for externalizing as a common mechanism of substance and behavioral‐based addiction. Results from this study call for the integration of problematic gambling, internet use, eating disorders and substance use under the umbrella of addiction and highlight the transdiagnostic value of externalizing outcomes across a range of psychopathology and developmental periods. This investigation is timely and imperative, given the increasing prevalence and recognition of behavioral addictions, yet the limited availability of GWAS evidence for these phenotypes.

## AUTHOR CONTRIBUTIONS

**Wei Q. Deng:** Conceptualization (lead); formal analysis (lead); investigation (lead); methodology (lead); project administration (lead); validation (equal); visualization (lead); writing—original draft (lead); writing—review and editing (lead). **Kyla Belisario:** Data curation (supporting); investigation (supporting); validation (equal); visualization (supporting); writing—original draft (supporting); writing—review and editing (equal). **Amanda Doggett:** Investigation (supporting); validation (equal); writing—review and editing (equal). **Marie Pigeyre:** Investigation (equal); validation (equal); writing—review and editing (equal). **Guillaume Pare:** Investigation (equal); methodology (supporting); writing—review and editing (equal). **Marcus R. Munafò:** Data curation (equal); funding acquisition (supporting); investigation (equal); writing—review and editing (equal). **James MacKillop:** Conceptualization (supporting); data curation (equal); funding acquisition (equal); investigation (equal); resources (lead); writing—review and editing (equal).

## DECLARATION OF INTERESTS

J.M. is a principal in Beam Diagnostics, and a consultant to Clairvoyant Therapeutics. No other authors have disclosures.

## ETHICS STATEMENT

Ethical approval for the study was obtained from the ALSPAC Ethics and Law Committee and the Local Research Ethics Committees. Informed consent for the use of data collected via questionnaires and clinics was obtained from participants following the recommendations of the ALSPAC Ethics and Law Committee at the time.

## Supporting information


**Table S1.** Description of all addiction‐related phenotypes curated from ALSPAC.
**Table S2.** Description of the final set of addiction‐related phenotypes for association analysis.
**Table S3.** Summary of the age range and time of collection for all addiction‐related phenotypes in ALSPAC.
**Table S4.** and details for the 22 sets of GWAS summary statistics.
**Table S5.** Prevalence and characteristics of key addiction‐related phenotypes in ALSPAC by sex and life stages.
**Table S6.** Pairwise associations between addiction‐related phenotypes and polygenic risk scores.
**Table S7.** Overlap of eating behavior variables associated with polygenic risk scores.
**Table S8.** A list of significant PRS‐by‐sex interactions between addiction‐related phenotypes and polygenic risk scores.
**Table S9.** A summary of linear and non‐linear trends in association strength between addiction‐related variables and polygenic risk scores over time.
**Table S10.** The 54 enriched pathways based on SNPs contribute to the PRS_EXT_.
**Table S11.** Pathway‐specific partition of PRS_EXT_ association with addiction‐related phenotypes.

**Figure S1.** A summary of pairwise correlations within addiction‐related phenotype categories.
**Figure S2.** A summary of pairwise correlations for PRSs.
**Figure S3.** A heatmap of pairwise association p‐values for all 22 PRSs.
**Figure S4.** Influence of sample size and effect size on association strength across phenotypic categories.
**Figure S5.** Venn Diagram of Eating Behavior Phenotypes Associated with PRSs.
**Figure S6.** Scatterplot of Effect Sizes with Confidence Intervals from Sensitivity Analysis.
**Figure S7.** A longitudinal trend of associations between selected polygenic risk scores and cannabis use phenotypes at multiple time points.
**Figure S8.** A longitudinal trend of associations between selected polygenic risk scores and tobacco use phenotypes at multiple time points.
**Figure S9.** A longitudinal trend of associations between selected polygenic risk scores and eating behavior phenotypes at multiple time points.
**Figure S10.** A longitudinal trend of associations between selected polygenic risk scores and gambling phenotypes at multiple time points.
**Figure S11.** A heatmap of pairwise association p‐values for pathway partitioned PRS_EXT_.
**Figure S12.** Summary of PRS_EXT_ Pathway Partitioned Associations Across Phenotypic Categories.

## Data Availability

Genotype and phenotype data are available through the ALSPAC data team pending a successful application. Access to the ALSPAC data was granted under project B3136. Polygenic risk scores generated in this study will be made available on the PGS catalog.
