# Supplementary material for: Externalizing as a common genetic influence for a broad spectrum of substance use and behavioral conditions: A developmental perspective from the Avon Longitudinal Study of Parents and Children
Source: Addiction. 2025 Aug 6;120(12):2559–72. doi: 10.1111/add.70163 (PMC12586799; doi:10.1111/add.70163)
Supplement: Supplementary file 1 — Table S1. Description of all addiction‐related phenotypes curated from ALSPAC. Table S2. Description of the final set of addiction‐related phenotypes for association analysis. Table S3. Summary of the age range and time of collection for all addiction‐related phenotypes in ALSPAC. Table S4. and details for the 22 sets of GWAS summary statistics. Table S5. Prevalence and characteristics of key addiction‐related phenotypes in ALSPAC by sex and life stages. Table S6. Pairwise associations between addiction‐related phenotypes and polygenic risk scores. Table S7. Overlap of eating behavior variables associated with polygenic risk scores. Table S8. A list of significant PRS‐by‐sex interactions between addiction‐related phenotypes and polygenic risk scores. Table S9. A summary of linear and non‐linear trends in association strength between addiction‐related variables and polygenic risk scores over time. Table S10. The 54 enriched pathways based on SNPs contribute to the PRSEXT. Table S11. Pathway‐specific partition of PRSEXT association with addiction‐related phenotypes. [file ADD-120-2559-s002.docx]

**Index of Supplementary Tables 1–11 (ordered by appearance in the main text)**

**Supplementary Table 1. Description of all addiction-related phenotypes curated from ALSPAC.**

<https://github.com/PBCAR/Project-Suppl-Files/blob/main/ALSPAC-phewas-2024/SuppTables-ALSPAC-PHEWAS-selected.xlsx>

**Supplementary Table 2. Description of the final set of addiction-related phenotypes for association analysis.**

<https://github.com/PBCAR/Project-Suppl-Files/blob/main/ALSPAC-phewas-2024/SuppTables-ALSPAC-PHEWAS-selected.xlsx>

**Supplementary Table 3. Summary of the age range and time of collection for all addiction-related phenotypes in ALSPAC.**

**Supplementary Table 4. References and details for the 22 sets of GWAS summary statistics.**


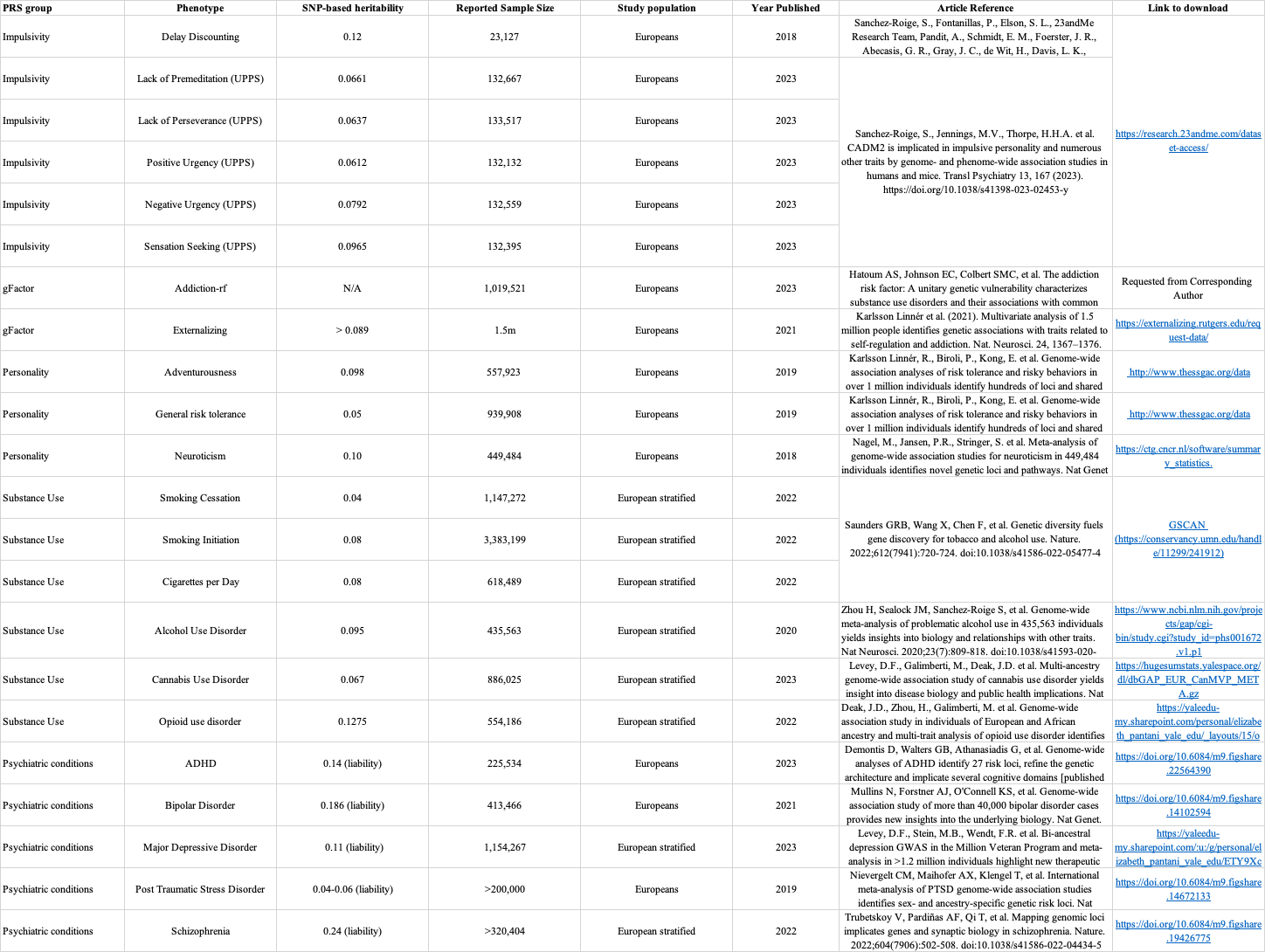


**Supplementary Table 5. Demographic and substance use characteristics of the ALSPAC G1 sample.**

**Supplementary Table 6. Pairwise associations between addiction-related phenotypes and polygenic risk scores.**

<https://github.com/PBCAR/Project-Suppl-Files/blob/main/ALSPAC-phewas-2024/SuppTables-ALSPAC-PHEWAS-selected.xlsx>

**Supplementary Table 7. Overlap of eating behavior variables associated with polygenic risk scores.**

**Supplementary Table 8. A list of significant PRS-by-sex interactions between addiction-related phenotypes and polygenic risk scores.**

**Supplementary Table 9.A summary of linear and non-linear trends in association strength between addiction-related variables and polygenic risk scores over time.**

**Supplementary Table 10. The 54 enriched pathways based on SNPs contribute to the PRSEXT.**

<https://github.com/PBCAR/Project-Suppl-Files/blob/main/ALSPAC-phewas-2024/SuppTables-ALSPAC-PHEWAS-selected.xlsx>

**Supplementary Table 11. Pathway-specific partition of PRSEXT association with addiction-related phenotypes.**
