# Supplementary material for: Externalizing as a common genetic influence for a broad spectrum of substance use and behavioral conditions: A developmental perspective from the Avon Longitudinal Study of Parents and Children
Source: Addiction. 2025 Aug 6;120(12):2559–72. doi: 10.1111/add.70163 (PMC12586799; doi:10.1111/add.70163)
Supplement: Supplementary file 2 — Figure S1. A summary of pairwise correlations within addiction‐related phenotype categories. Figure S2. A summary of pairwise correlations for PRSs. Figure S3. A heatmap of pairwise association p‐values for all 22 PRSs. Figure S4. Influence of sample size and effect size on association strength across phenotypic categories. Figure S5. Venn Diagram of Eating Behavior Phenotypes Associated with PRSs. Figure S6. Scatterplot of Effect Sizes with Confidence Intervals from Sensitivity Analysis. Figure S7. A longitudinal trend of associations between selected polygenic risk scores and cannabis use phenotypes at multiple time points. Figure S8. A longitudinal trend of associations between selected polygenic risk scores and tobacco use phenotypes at multiple time points. Figure S9. A longitudinal trend of associations between selected polygenic risk scores and eating behavior phenotypes at multiple time points. Figure S10. A longitudinal trend of associations between selected polygenic risk scores and gambling phenotypes at multiple time points. Figure S11. A heatmap of pairwise association p‐values for pathway partitioned PRSEXT. Figure S12. Summary of PRSEXT Pathway Partitioned Associations Across Phenotypic Categories. [file ADD-120-2559-s001.docx]

Supplementary Materials for “ALSPAC Phewas”

Table of Contents

[Phenotype curation and processing 2](#_Toc184990988)

[Alcohol use 2](#_Toc184990989)

[Smoking 2](#_Toc184990990)

[e-cig use 3](#_Toc184990991)

[Cannabis use 3](#_Toc184990992)

[Gambling 4](#_Toc184990993)

[Eating behaviour 4](#_Toc184990994)

[Internet use 5](#_Toc184990995)

[Polygenic risk score construction 5](#_Toc184990996)

[Pathway partitioned PRS analysis 6](#_Toc184990997)

[Table and Figure Legends 6](#_Toc184990998)

[REFERENCE: 12](#_Toc184990999)

# Phenotype curation and processing

Phenotypes were curated from a successful data application under project # B3136 titled “Impulsivity as a Risk Mechanism in the ALSPAC Cohort Longitudinal Augmentation”. Thus, we have full access to variables for impulsivity (delay discounting, UPPS-P questionnaire) and the majority of substance use variables, however, certain variables for eating behaviours are unavailable, for example, at the CCU wave. For substance use phenotypes, we curated phenotypes on quantity and frequency of use, age of initiation when possible, as well as well-established screening tests. For behavioural-based addition phenotypes, we took a more holistic approach and did not intentionally remove variables except when the variable was deemed non-informative (case count <= 2 or singular). Frequency and quantity phenotypes over time were consolidated to have the same ordering and numerical codes.

## Alcohol use

**Alcohol use frequency** was assessed using the Alcohol Use Disorder Identification Test (AUDIT). The first three items were first re-coded (0–3) before being summed to create an overall score. Those not endorsing alcohol use were given an AUDIT score of 0.

**Problematic alcohol use** was also assessed using AUDIT. The last 7 items were re-coded (0–3) before being summed to create an overall score. Those not endorsing alcohol use were given a score of 0.

## Smoking

**Age of initiation** was consolidated across multiple time points and a non-missing value was only assigned to those who indicated that they ever smoked a whole cigarette. The most consistently recalled minimum age for the first smoking was 7 and thus a missing value was given to those with age initiation below 7.

**Current smoker status** was defined for those who indicated to be weekly smokers. Several quality control variables were used to confirm current weekly smoking, including ‘ever’ status and last smoked in the past 30 days. Those who had conflicting statuses or only indicated to not have smoked in the last 30 days were assigned non-current smoking status. This protocol ensures that those who do not currently smoke but did not answer the question would be correctly imputed.

**Cigarettes per day** were collapsed from the weekly and daily number of cigarettes for those who indicated to be weekly and daily smokers, respectively. The daily number was prioritized and then those missing daily usage were imputed from the weekly usage. Those who indicated to be current non-smokers were assigned a value of 0 for the number of cigarettes smoked daily.

**Nicotine dependence** was calculated using the brief Fagerström Test for Nicotine Dependence (FTND; Heatherton et al., 1991). The number of cigarettes consumed per day was used for item 4 on the FTND, and the remaining FTND items were re-coded according to the scoring methods outlined (Heatherton et al., 1991). Individuals not endorsing daily smoking were not administered the FTND, and subsequently they were given an FTND score of zero.

## e-cig use

**Age of initiation** was consolidated across the two time points for which e-cig use questions were implemented at YPC (age 23) and YPD (age 24), for which a non-missing value was assigned to those who indicated that they ever used an e-cigarette or other vaping device.

**Ever use status** was based on participants indicating ever used/vaped an electronic cigarette (e-cigarette) or other vaping device.

**Current use status** was based on participants indicating currently uses/vape an electronic cigarette (e-cigarette) or other vaping device.

**Current use frequency** was based on participants indicating the frequency of using/vaping an electronic cigarette (e-cigarette) or other vaping device. The most frequent option is “At least once a day”, which was coded to be 4, then “At least once a week”, which received a coding of 3, and “At least once a month” and “Less than once a month” were coded as 2 and 1, respectively. Those who indicated ever use but not currently using were assigned a value of 0.

**Duration of use** was a unique phenotype collected on e-cig use, indicating the length of time during which the participant has used e-cigarettes/vaping devices. The context of the question was to understand e-cig usage habit in young adults as the popularity of e-cig become apparent. The options were “<1 month”, “1–3 months”, “4–6 months”, “7 months to 1 year”, “1–2 years”, or “>2 years”, and coded as 1, 2, 3, 4, 5, 6, respectively. Those who indicated ever use but not currently using were removed.

**Millilitres of liquid used** was another unique variable to measure quantity of consumption for e-cig due to its construction. It measures how much of the refillable electronic cigarette device was used by the respondent on an average day. The ml quantity was coded by integers 1 to 7 incrementally, corresponding to “<1 ml”, “1–2ml” , “2–4ml” , “4–6ml” , “6–8ml” , “8–10ml” , “>10ml”. Those who indicated ever use but not currently using were assigned a value of 0.

## Cannabis use

**Age of initiation** was consolidated across multiple time points and a non-missing value was only assigned to those who indicated that they ever consumed cannabis.

**Cannabis ever use status** was defined for participants indicating ever tried cannabis (also called marijuana, hash, dope, pot, blow, skunk, puff, grass, draw, ganja, joints, smoke, weed).

**Cannabis use frequency** was based on self-report on usage in the past year. The most frequent option is “Daily or almost daily”, which was coded to be 5, then “Weekly”, which received a coding of 4, and “Monthly” and “Less than monthly” were coded as 3 and 2, respectively. Those who indicated occasional use (“once or twice”) were assigned a value of 1. Those who indicated to have never used cannabis were removed.

**Problematic cannabis use** was assessed using the Cannabis Abuse Screening Test (CAST; Legleye, 2017). The 6 items were first re-coded as outlined by the scale of Legleye (2017) before being summed to create an overall score. Those not endorsing cannabis use were given a CAST score of 0.

## Gambling

**Gambling status** was defined as by participation in one of the 17 activities (See below) at least once in the past 12 months.

**Gambling frequency** was available for a total of 17 activities that were have gambling in nature, including 1) National Lottery (not including scratchcards), 2) National Lottery or online scratchcards (not including newspaper or magazine scratchcards), 3) tickets for any 'other' lottery not including Irish or international lotteries or raffle tickets, 4) football pools, 5) bingo cards or tickets not including newspaper bingo tickets or online bingo, 6) fruit slot machines, not including quiz machines, 7) virtual gaming machines in a bookmakers to bet on Roulette, Keno bingo, etc not including quiz machines, 8) table games (roulette, dice or cards) in a casino, 9) on-line gambling like playing poker, bingo, slot machine style games, or casino games 'for money' not including on-line bets with bookmakers or betting exchanges, 10) on-line betting 'with a bookmaker' on any event or sport not including exchange or spread betting, 11) Betting Exchange, 12) horses in a bookmakers, by phone or at the track not including exchange betting, 13) dogs in a bookmakers, by phone or at the track not including on-line bets with bookmakers or betting exchanges, 14) bets on other events at a bookmakers, by phone or at the venue not including on-line bets with bookmakers, betting exchanges or spread betting, 15) spread-betting, 16) private betting, playing cards or games for money with friends, family or colleagues, 17) any other form of gambling, in the last 12 months. Those who indicated never gambled were assigned a value of 0.

**Problematic gambling** was assessed using the 9 items from the Problem Gambling Severity Index (PGSI; (Ferris & Wynne, 2001)). Each item was first re-coded to 1–4, corresponding to “never”, “sometimes”, “most of the time”, and “almost always”, before being summed to create an overall score. Those who indicated never gambled in the past 12 months were assigned a value of 0.

## Eating behaviour

A range of eating behaviours, including binge eating, purging, excessive exercise, fasting, and self-perception of body image, across four time points (13 (“TA”), 15 (“TC”), 18 (“CCT”), 24(“YPD”)) and professionally diagnosed binge eating disorder, anorexia nervosa, and bulimia nervosa for the last time point (“YPD”), were included.

## Internet use

There were relatively fewer questions on internet use, we retained questions related to the number of hours spent online per week for activities ranging from information search, socializing, leisure, e-commerce at age 18 (“CCXC”), frequency of gaming and parental restriction due to internet use at age 13 (“TA”), and hours spent using electronics (including computer/phone/tablet/e-book) on an average weekday and weekend at age 22 (“YPB”) and age 26 (“YPF”).

# Polygenic risk score construction

In addition to the genetic QCs, we performed additional QC steps to ensure the reference alleles matched between each discovery GWAS and the target genotype data from ALSPAC. PRSs were derived using Lassosum (Mak et al., 2017) for its versatility and robustness to misspecification of linkage equilibrium (LD).

One of the key challenges was to derive validating phenotypes to optimize the SNP weights for the final PRS. We outlined these phenotypes in Suppl. Table 3 in which we made our decision based on 1) the availability of the exact or a close match of the discovery GWAS phenotype; and 2) the sample size for the available validating phenotype, for example, we decided to not validate Neuroticism with neurotic symptom score at F17 as only 1,757 samples were available; and 3) if the case count or variability of the validating phenotype was deemed too low (< 5%) to produce an effective optimization. For phenotypes with multiple matches at different time points, we prioritized based on sample size and the most recently available measure.

As a result, PRSs for all psychiatric conditions, AUD, PAU, CUD, and OUD, were constructed using the pseudo-validation option, in which only the discovery GWAS and LD patterns in the ALSPAC genotypes were used to derive the final PRSs. PRSs of externalizing and general addiction were also based on pseudo-validation as they reflect the broad genetic constructs encompassing multiple traits.

For in-sample performance, we calculated the adjusted R^2^ and the p-value associated with PRS in the linear regression model with the validating phenotype as the outcome, adjusting for all covariates. We allowed a more flexible definition of proxy phenotypes to evaluate the performance of the derived PRSs. Further, a less stringent p-value threshold (*p* < 0.05) was used to determine whether a PRS should be retained for further analysis.

# Pathway partitioned PRS analysis

Here we focused on a single PRS as an example to demonstrate the workflow for the partitioned PRS approach. First, we obtained the list of selected SNPs and their associated weights from the internal output of the lassosum PRS construction. The number of SNPs retained depends on the underlying genetic architecture of the trait. Second, we then mapped these SNPs to the nearest gene within 200Kilobases distance from the SNP using VEP (https://grch37.ensembl.org/Homo_sapiens/Tools/VEP; accessed on August 26, 2024). If one SNP was mapped to the multiple genes within this region, we retained the nearest gene with an Ensembl ID. Third, the mapped genes (after removing duplicates) were then used to test for enrichment in KEGG pathways (Kanehisa et al., 2022) using a false discovery rate adjusted *p*-value cut-off of 0.01, producing pathways in seven broad categories, including metabolism, genetic information processing, environmental information processing, cellular processes, organismal systems, human diseases, and drug development. The pathways represent the state of our knowledge of molecular interaction, reaction, and relation networks. Since the same gene can be mapped to multiple pathways, each SNP (through genes) can contribute to the pathway-specific PRS more than once. Finally, we partitioned the PRS by SNPs assigned to individually enriched pathways. These pathway-specific PRSs were then tested for association with addiction-related phenotypes as described previously.

# Table and Figure Legends

**Table S1 Description of all addiction-related phenotypes curated from ALSPAC.**

**Table S2 Description of the final set of addiction-related phenotypes for association analysis.**

**Table S3 Summary of the age range and time of collection for all addiction-related phenotypes in ALSPAC.**

**Table S4 and details for the 22 sets of GWAS summary statistics**

**Table S5 Prevalence and characteristics of key addiction-related phenotypes in ALSPAC by sex and life stages.**

**Table S6 Pairwise associations between addiction-related phenotypes and polygenic risk scores.**

**Table S7 Overlap of eating behavior variables associated with polygenic risk scores.**

**Table S8 A list of significant PRS-by-sex interactions between addiction-related phenotypes and polygenic risk scores.**

**Table S9 A summary of linear and non-linear trends in association strength between addiction-related variables and polygenic risk scores over time.**

**Table S10 The 54 enriched pathways based on SNPs contribute to the PRS_EXT_.**

**Table S11 Pathway-specific partition of PRS_EXT_ association with addiction-related phenotypes.**

**Figure S1. A summary of pairwise correlations within addiction-related phenotype categories.**

Heatmaps were used to visualize the dynamic patterns of addiction-related phenotypes over time, providing insights into changes in severity and frequency. Heatmap showing substance use (1-A), eating behaviors (1-B), gambling behaviors (1-C), and internet use (1-D) phenotypes across multiple time points. The color intensity represents the strength of correlation between pairwise relationships, with blue indicating a strong negative correlation and red indicating a strong positive correlation coefficient.

**Figure S2. A summary of pairwise correlations for PRSs.**

The heatmap illustrates the pairwise correlations between different Polygenic Risk Scores (PRSs). The color intensity represents the strength and direction of the correlations, with warmer colors (e.g., red) indicating stronger positive correlations and cooler colors (e.g., blue) representing stronger negative correlations. Each cell corresponds to the correlation between two PRSs, and the color gradient provides a visual scale of the correlation coefficients. The labels on the x- and y-axes identify the individual PRSs, arranged based on PRSs for impulsivity, general factors, personality, substance use, and psychiatric conditions.

**Figure S3. A heatmap of pairwise association p-values for all 22 PRSs.**

The heatmap summarizes the strength of associations between Polygenic Risk Scores (PRSs) and phenotypes in four categories. The color scale represents the -log10(p-value) of the associations, with darker red indicating stronger associations (higher -log10(p-value)) and white representing no association. The x-axis lists the phenotype categories, while the y-axis represents the individual PRSs. This heatmap visually highlights areas of significant association, with deeper shades of red corresponding to higher statistical significance.


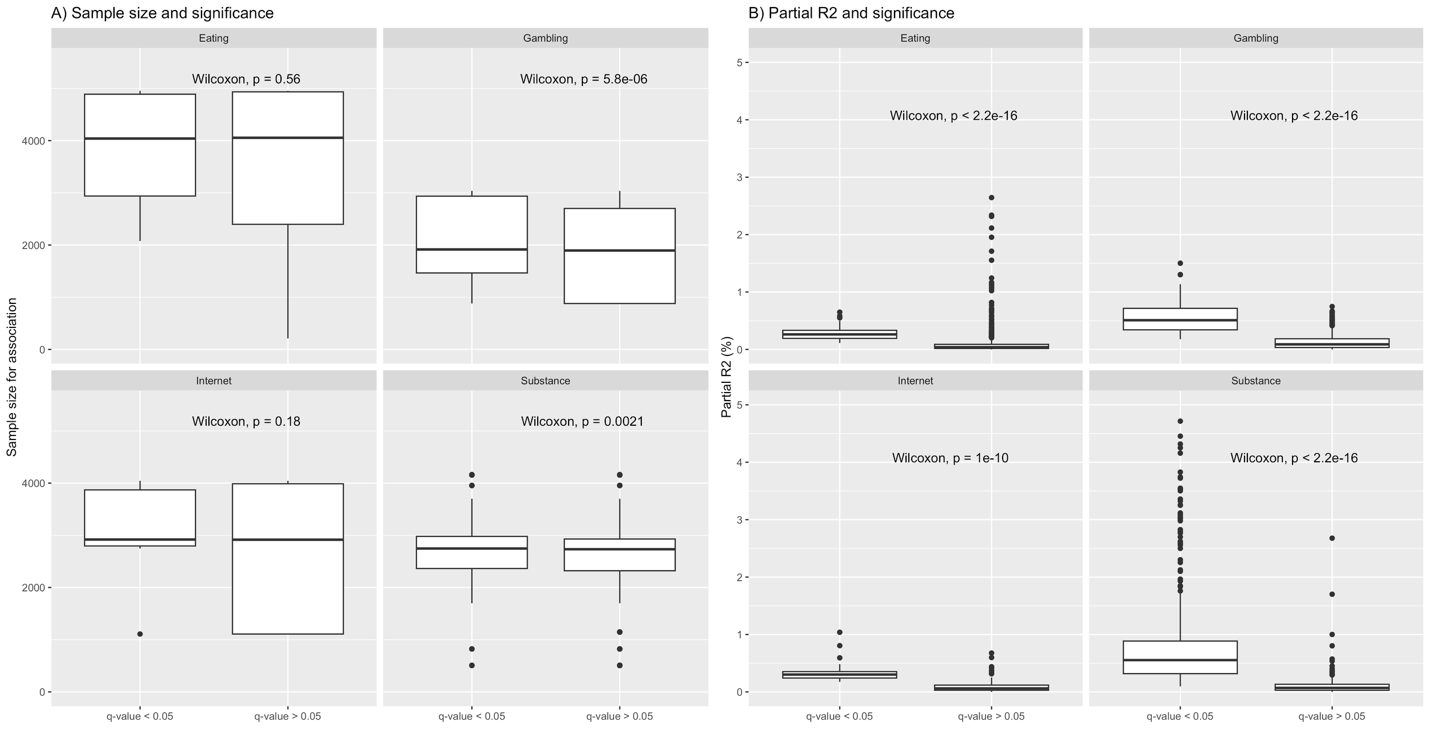


**Figure S4. Influence of sample size and effect size on association strength across phenotypic categories.**

We examined the influence of sample size and estimated effect size on the significance of results across four phenotypic categories. In some cases, not all phenotypic data could be collected for every participant, leading to variability in sample sizes across different categories. This can result in uneven representation of samples within each phenotypic category, especially when stratifying by significance thresholds ( q < 0.05 vs. q > 0.05 ). As a result, associations observed in some categories may involve smaller or larger sample sizes depending on the availability of data and could bias results towards phenotypes that had larger sample sizes. In A), the boxplots compare the sample size stratified by significance thresholds. For each phenotypic category, the left boxplot shows the range of sample sizes for associations where q < 0.05, while the right boxplot displays the sample sizes for associations where q > 0.05. In B), the second set of boxplots compares the partial R^2^ values, also stratified by significance thresholds. For each phenotypic category, the left boxplot represents the partial R^2^ for associations with q < 0.05, while the right boxplot shows the partial R^2 for associations with q > 0.05. The Wilcoxon rank-sum test (or Mann-Whitney U test) was used to assess whether the distributions of sample size and partial R^2 between associations with q < 0.05 and > 0.05 differ significantly.

**Figure S5. Venn Diagram of Eating Behavior Phenotypes Associated with PRSs.**

The Venn Diagram illustrates the overlap of eating behavior phenotypes associated with four Polygenic Risk Scores (PRSs). Each circle represents a different PRS, and the areas where the circles overlap indicate shared phenotypic associations between multiple PRSs. The diagram highlights both unique and common associations across the PRSs, providing a visual summary of how different genetic risk scores are linked to specific eating behavior phenotypes. The color of each section corresponds to the number of significant associations observed for each PRS or their combinations, with darker red corresponding to a higher count and white corresponding to a zero count.


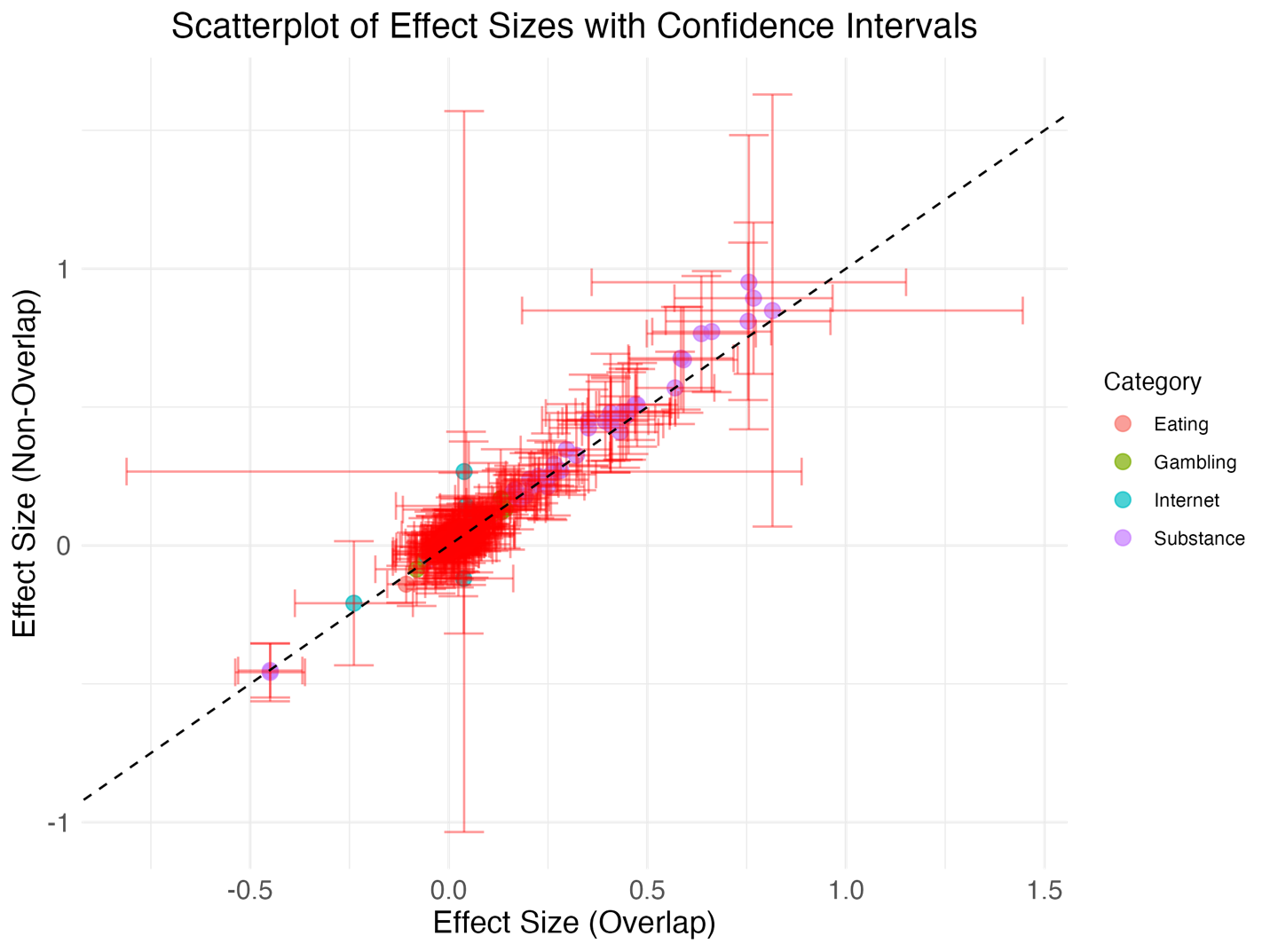


**Figure S6. Scatterplot of Effect Sizes with Confidence Intervals from Sensitivity Analysis**

PRS_EXT_ association effect sizes from the same regression models comparing ALSPAC samples with discovery-target overlap (x-axis) versus samples without overlap (y-axis). Each point represents a addiction-related outcome across four categories: eating behaviors (red), gambling behaviors (green), internet use behaviors (cyan), and substance use behaviors (purple). Error bars show 95% confidence intervals. The dashed line represents perfect correlation (y = x). Points clustering along the diagonal indicate consistent effect sizes between overlapping and non-overlapping samples, demonstrating minimal bias from sample overlap (R² = 0.99).


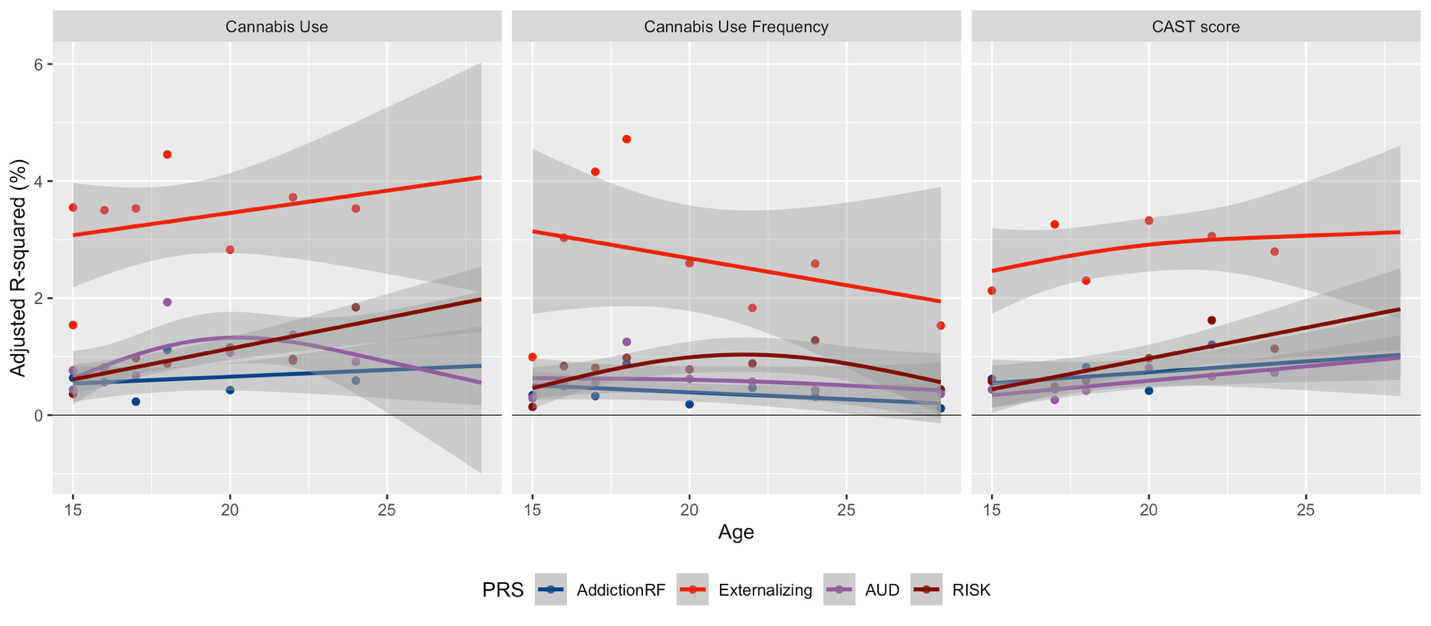


**Figure S7. A longitudinal trend of associations between selected polygenic risk scores and cannabis use phenotypes at multiple time points.**

The association strength (adjusted R^2^) was shown as a function of the time when the phenotype was measured. The three panels corresponded to results based on cannabis use, use frequency, and severity. Each dot represented the estimated adjusted R^2^ using the PRS_addiction-rf_, PRS_EXT_, PRS_AUD_, and PRS_RISK_. The best-fitted line was estimated using the Generalized Additive Models with smoothness determined by 3 degrees of freedom. The shaded area around the fitted line represents the 95% confidence interval, estimated using bootstrap sampling techniques.


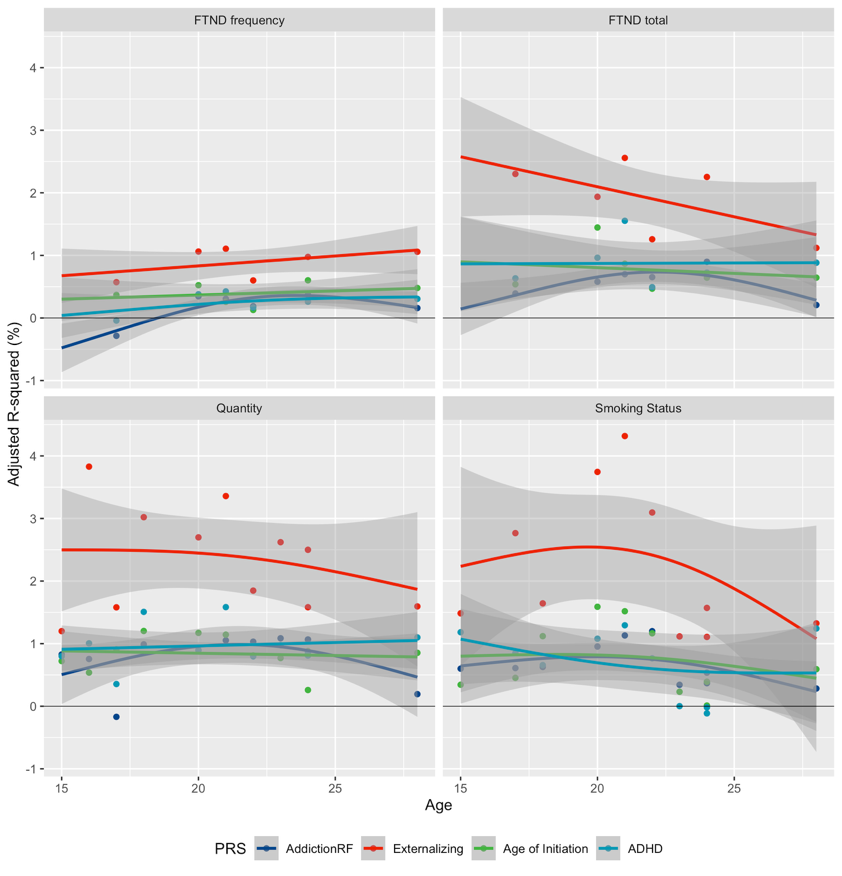


**Figure S8. A longitudinal trend of associations between selected polygenic risk scores and tobacco use phenotypes at multiple time points.**

The association strength (adjusted R^2^) was shown as a function of the time when the phenotype was measured. The four panels corresponded to results based on FTND frequency, FTND total score, quantity of cigarettes smoked, and smoking status. Each dot represented the estimated adjusted R^2^ using the PRS_addiction-rf_, PRS_EXT_, PRS_SI_, and PRS_ADHD_. The best-fitted line was estimated using the Generalized Additive Models with smoothness determined by 3 degrees of freedom. The shaded area around the fitted line represents the 95% confidence interval, estimated using bootstrap sampling techniques.

**
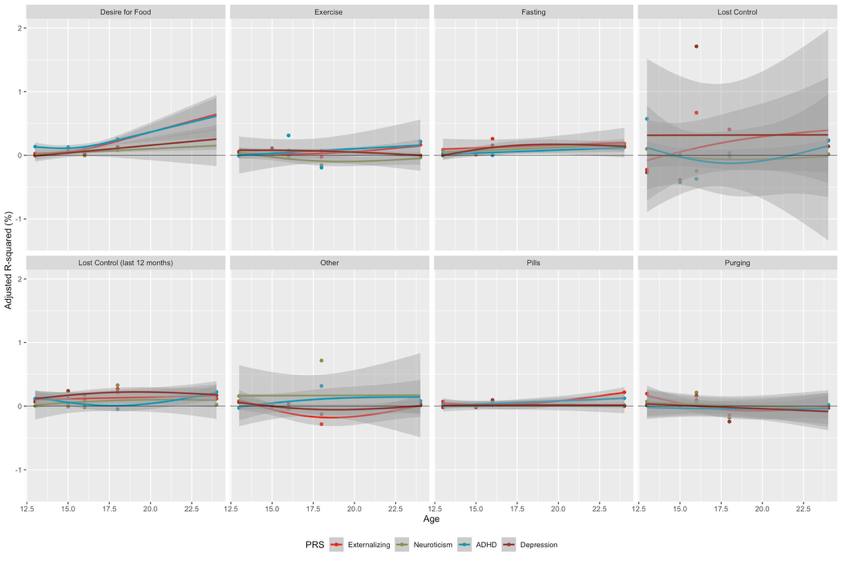
**

**Figure S9. A longitudinal trend of associations between selected polygenic risk scores and eating behavior phenotypes at multiple time points.**

The association strength (adjusted R^2^) was shown as a function of the time when the phenotype was measured. The panels corresponded to results under different types of behaviors, including desire for food, excessive exercise, fasting, a sense of lost control, a sense of lost control in the last 12 months, taking pills, purging, and other. Each dot represented the estimated adjusted R^2^ using the PRS_ADHD_, PRS_EXT_, PRS_Neuroticism_, and PRS_MDD_. The best-fitted line was estimated via the Generalized Additive Models with smoothness determined by 3 degrees of freedom. The shaded area around the fitted line represents the 95% confidence interval, estimated using bootstrap sampling techniques.

**
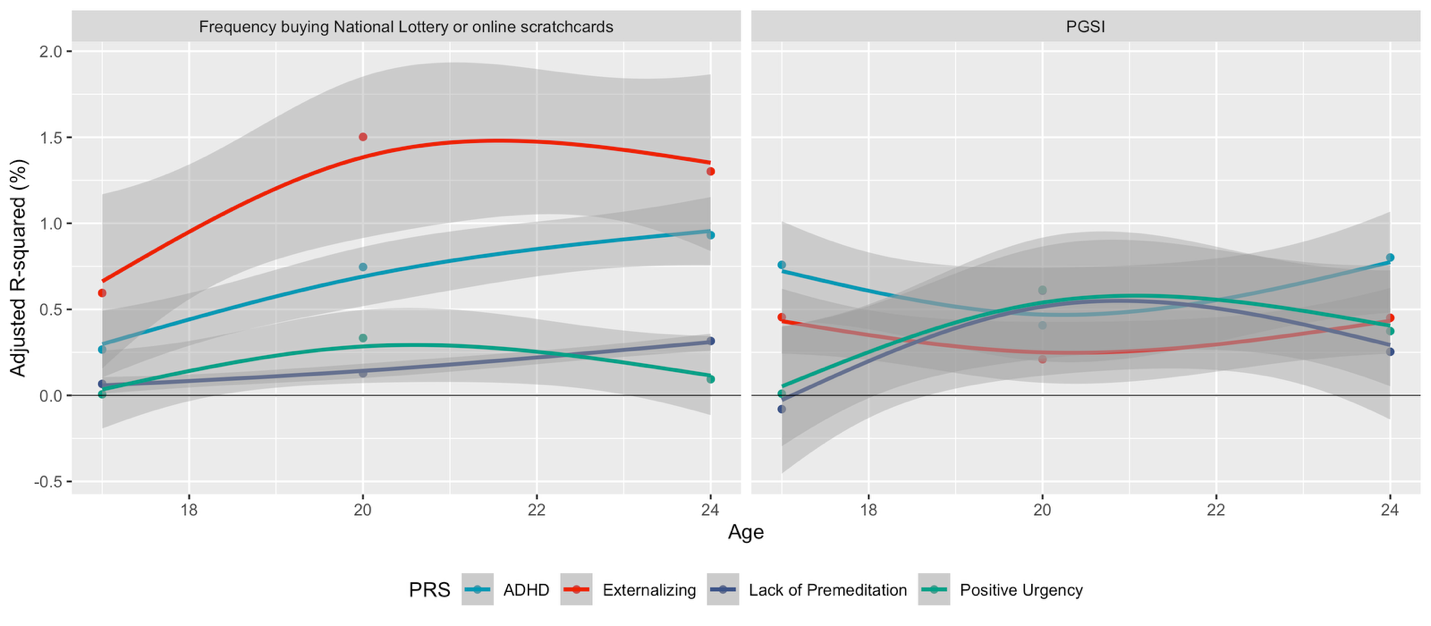
**

**Figure S10. A longitudinal trend of associations between selected polygenic risk scores and gambling phenotypes at multiple time points.**

The association strength (adjusted R^2^) was shown as a function of the time when the phenotype was measured. The three panels corresponded to results based on gambling frequency of national lottery, and the Problem Gambling Severity Index total score. Each dot represented the estimated adjusted R^2^ using the PRS_ADHD_, PRS_EXT_, PRS_UPPS-PreMed_, and PRS_UPPS-PU_. The best-fitted line was estimated using the Generalized Additive Models with smoothness determined by 2 degrees of freedom. The shaded area around the fitted line represents the 95% confidence interval, estimated using bootstrap sampling techniques.

**Figure S11. A heatmap of pairwise association p-values for pathway partitioned PRS_EXT_.**

The heatmap summarizes the strength of associations between pathway-partitioned PRS_EXT_ and phenotypes in four categories. The color scale represents the -log10(p-value) of the associations, with blue indicating stronger associations (higher -log10(p-value)) and white representing no association. The x-axis lists the phenotype categories, while the y-axis represents the individual pathways. This heatmap visually highlights areas of significant association, with deeper shades of red corresponding to higher statistical significance.

**Figure S12. Summary of PRS_EXT_ Pathway Partitioned Associations Across Phenotypic Categories**

Bar plots displaying the number of associations across four categories (Substance use, Gambling, Eating behaviors, and Internet use) for the 21 pathways (partitioned PRS_EXT_ )associated with at least one phenotype. The height of each bar represents the total number of associations for a given pathway partition, with different colors indicating the contribution of each category to the overall total. The y-axis denotes the number of associations, and the x-axis lists the individual pathways.

# REFERENCE:

Ferris, J., & Wynne, H. (2001). The Canadian Problem Gambling Index : Final report. In *Canadian Centre on Substance Abuse*.

HEATHERTON, T. F., KOZLOWSKI, L. T., FRECKER, R. C., & FAGERSTROM, K. ‐O. (1991). The Fagerström Test for Nicotine Dependence: a revision of the Fagerstrom Tolerance Questionnaire. *British Journal of Addiction*, *86*(9). https://doi.org/10.1111/j.1360-0443.1991.tb01879.x

Kanehisa, M., Sato, Y., & Kawashima, M. (2022). KEGG mapping tools for uncovering hidden features in biological data. *Protein Science*, *31*(1). https://doi.org/10.1002/pro.4172

Kolberg, L., Raudvere, U., Kuzmin, I., Adler, P., Vilo, J., & Peterson, H. (2023). G:Profiler-interoperable web service for functional enrichment analysis and gene identifier mapping (2023 update). *Nucleic Acids Research*, *51*(W1). https://doi.org/10.1093/nar/gkad347

Mak, T. S. H., Porsch, R. M., Choi, S. W., Zhou, X., & Sham, P. C. (2017). Polygenic scores via penalized regression on summary statistics. *Genet. Epidemiol.*, *41*(6), 469–480. https://doi.org/10.1002/gepi.22050
